# Error-related brain activity as a transdiagnostic endophenotype for obsessive-compulsive disorder, anxiety and substance use disorder

**DOI:** 10.1017/S0033291719000199

**Published:** 2019-02-12

**Authors:** Anja Riesel, Julia Klawohn, Rosa Grützmann, Christian Kaufmann, Stephan Heinzel, Katharina Bey, Leonhard Lennertz, Michael Wagner, Norbert Kathmann

**Affiliations:** 1 Department of Psychology, Humboldt-Universität zu Berlin, Berlin, Germany; 2 Departments of Biomedical Sciences and Psychology, Florida State University, Tallahassee, FL, USA; 3 Department of Psychology, Freie University Berlin, Berlin, Germany; 4 Department of Psychiatry and Psychotherapy, University of Bonn, Bonn, Germany; 5 German Center for Neurodegenerative Diseases (DZNE), Bonn, Germany

**Keywords:** Anxiety disorders, depression, endophenotype, error-related negativity, family study, obsessive-compulsive disorder, substance use disorder, transdiagnostic risk marker, unaffected first-degree relatives

## Abstract

**Background:**

Increased neural error-signals have been observed in obsessive-compulsive disorder (OCD), anxiety disorders, and inconsistently in depression. Reduced neural error-signals have been observed in substance use disorders (SUD). Thus, alterations in error-monitoring are proposed as a transdiagnostic endophenotype. To strengthen this notion, data from unaffected individuals with a family history for the respective disorders are needed.

**Methods:**

The error-related negativity (ERN) as a neural indicator of error-monitoring was measured during a flanker task from 117 OCD patients, 50 unaffected first-degree relatives of OCD patients, and 130 healthy comparison participants. Family history information indicated, that 76 healthy controls were free of a family history for psychopathology, whereas the remaining had first-degree relatives with depression (*n* = 28), anxiety (*n* = 27), and/or SUD (*n* = 27).

**Results:**

Increased ERN amplitudes were found in OCD patients and unaffected first-degree relatives of OCD patients. In addition, unaffected first-degree relatives of individuals with anxiety disorders were also characterized by increased ERN amplitudes, whereas relatives of individuals with SUD showed reduced amplitudes.

**Conclusions:**

Alterations in neural error-signals in unaffected first-degree relatives with a family history of OCD, anxiety, or SUD support the utility of the ERN as a transdiagnostic endophenotype. Reduced neural error-signals may indicate vulnerability for under-controlled behavior and risk for substance use, whereas a harm- or error-avoidant response style and vulnerability for OCD and anxiety appears to be associated with increased ERN. This adds to findings suggesting a common neurobiological substrate across psychiatric disorders involving the anterior cingulate cortex and deficits in cognitive control.

## Introduction

Obsessive-compulsive disorder (OCD) is characterized by intrusive obsessions and repetitive compulsions. The lifetime prevalence is estimated to be 1–3% (Ruscio *et al*., 2010). Despite its prevalence and its impact on life quality, the pathophysiology of OCD is not well understood. Further, despite accumulating evidence for heritability of OCD and associated traits (Pauls *et al*., 2014), reliable evidence for the involvement of specific genes has not emerged (Pauls *et al*., 2014; International Obsessive Compulsive Disorder Foundation Genetics and Studies, 2017). OCD is etiologically complex, with multiple genetic, epigenetic, and environmental factors as well as their interactions contributing to its development (Pauls *et al*., 2014). Phenotypic heterogeneity, overlap in symptoms, and high comorbidity between psychiatric disorders (Insel *et al*., 2010; Ruscio *et al*., 2010) further complicate research and it remains a major scientific challenge to unravel the etiology of complex disorders such as OCD. The Research Domain Criteria (RDoC) initiative and the endophenotype concept are hoped to help overcoming these problems (Gottesman and Gould, 2003; Insel *et al*., 2010; Miller and Rockstroh, 2013). RDoC takes a transdiagnostic perspective and conceptualizes psychopathology with a stronger grounding in neuroscience and in relation to dysfunctions in fundamental dimensions of behavior and neurobiology (Insel *et al*., 2010). Endophenotypes are heritable biological or psychological traits that represent simpler signs to etiological underpinnings and are assumed to be closer to genes and disease mechanisms (Gottesman and Gould, 2003; Insel *et al*., 2010; Miller and Rockstroh, 2013). Both concepts consistently emphasize a grounding in neuroscience, a focus on disease mechanisms, and transdiagnostic processes (Miller and Rockstroh, 2013).

Neural error-signals are a promising research target within both concepts, and alterations in error-processing are suggested to play a role in several mental disorders. The error-related negativity (ERN) is a well-validated and established electrophysiological marker of error-processing. The ERN is a negative deflection in the event-related brain potential peaking over fronto-central electrodes following an error and has been associated with activity in the midcingulate cortex predominantly the anterior cingulate cortex (ACC; Debener *et al*., 2005; Manoach and Agam, 2013; Grutzmann *et al*., 2016).^†^^1^ The ERN has been repeatedly found to be increased in OCD patients (Endrass and Ullsperger, 2014; Riesel, 2019) and fulfills key criteria for an endophenotype (Gottesman and Gould, 2003; Miller and Rockstroh, 2013), including heritability (Anokhin *et al*., 2008), and a robust association with the disorder (Endrass and Ullsperger, 2014; Riesel, 2019). Furthermore, increased ERN amplitudes persist after symptom reduction over the course of cognitive behavioral therapy (CBT, Hajcak *et al*., 2008; Huyser *et al*., 2011; Riesel *et al*., 2015) and are also observed in unaffected first-degree relatives of OCD patients in the absence of symptoms (Riesel *et al*., 2011; Carrasco *et al*., 2013). Altogether, these results highlight that increased ERN amplitudes seem not to be the consequence or a correlate of OCD symptoms but to rather represent a promising endophenotype reflecting vulnerability for the disorder.

However, increased ERN amplitudes are not specific to OCD. Hyperactive neural error-signals in the brain have been linked to worry (Moser *et al*., 2013), repetitive behavior (Manoach and Agam, 2013), checking (Weinberg *et al*., 2015*b*), and anxiety proneness (Cavanagh and Shackman, 2014), symptoms that represent core features of OCD but are shared with other disorders. In the same vein, increased ERN amplitudes are not specific to OCD but have also been observed in generalized anxiety disorder (GAD), social anxiety, health anxiety, and less consistently in depression (Manoach and Agam, 2013; Endrass *et al*., 2014; Weinberg *et al*., 2015*a*; Gillan *et al*., 2017; Riesel *et al*., 2017). Moreover, increased amplitudes prospectively predict the development of anxiety symptoms (Lahat *et al*., 2014; Lamm *et al*., 2014; Meyer *et al*., 2015; Meyer *et al*., 2018). In contrast, reduced ERN amplitudes have been found in schizophrenia, bipolar disorder (Minzenberg *et al*., 2014), substance use disorder (SUD) and, inconsistently, in autism and attention-deficit/hyperactivity disorder (Manoach and Agam, 2013; Luijten *et al*., 2014; Gillan *et al*., 2017). In schizophrenia and SUD, results from a treatment study and high-risk populations indicate that error-processing deficits precede illness onset (Simmonite *et al*., 2012; Euser *et al*., 2013; Manoach and Agam, 2013; Gillan *et al*., 2017). Against this background, error-related brain activity qualifies as a promising transdiagnostic endophenotype with both reduction and enhancement in neural error-signals each indicating vulnerability for different types of psychopathology. To further strengthen this notion, studies examining unaffected first-degree relatives are of crucial importance given that family history is a major risk-factor for the development of psychopathology. The current study aims to replicate the finding that OCD patients and their unaffected first-degree relatives show increased ERN amplitudes. Moreover, we investigate whether alterations in neural error-signals reflect vulnerability across diagnoses by examining unaffected first-degree relatives of individuals with anxiety disorders, depression, and SUD. We hypothesize that individuals with familial risk for anxiety show increased ERN amplitudes, whereas those at risk for SUD show reduced amplitudes.

## Methods

### Participants

One hundred and seventeen patients with OCD, 50 unaffected first-degree relatives of patients with OCD (parents: *n* = 34, siblings: *n* = 13, offspring: *n* = 3), and 130 healthy comparison participants without a family history of OCD participated in the study (see Table 1). All participants were examined by trained clinical psychologists using the Structured Clinical Interview for Diagnostic and Statistical Manual of Mental Disorders (DSM)-IV (SCID, First *et al*., 1996) to assess past and present psychiatric disorders. For all participants family history information of psychopathology for their first-degree relatives was collected using the Family History Screen (Weissman *et al*., 2000), a structured interview that assesses all major DSM-IV diagnoses. Of the 130 healthy comparison participants, 76 were free of any family history for psychopathology (i.e. none of their first-degree relatives fulfilled criteria for a major DSM diagnosis), whereas the remaining reported a family history of depression (*unaffected first-degree relatives of individuals with depression*, *n* = 28), anxiety disorders (*unaffected first-degree relatives of individuals with anxiety disorders*, *n* = 27), and/or SUD (*unaffected first-degree relatives of individuals with SUD*, *n* = 27)^2^. All participants were between 18 and 65 years of age, had normal or corrected-to-normal vision, and reported no history of neurological disease or head trauma. Details about the subject flow and dropout reasons are presented in the supplementary information (online Supplementary Fig. 1).

**Table 1.** Demographic and clinical characteristics and performance data of patients with OCD, unaffected first-degree relatives, and unaffected comparison subjects

	Patients with OCD		Unaffected first-degree relatives of OCD patients		Healthy comparison participants		Statistic	*p*
	Mean	s.d.	Mean	s.d.	Mean	s.d.		
Demographical data								
*N*	117		50		130			
Mean age, years	31.9	9.4	45	14.8	32.1	9.9	*F*_(2, 294)_ = 30.22	0.00
Gender (% female)	53		64		58		χ^2^(2) = 1.79	0.41
Verbal IQ	103.2	10	106.5	9.6	104.8	9.4	*F*_(2, 294)_ = 2.17	0.12
Family history								
Depressive disorders *n* in %	45.3		54		21.5		χ^2^(2) = 30.44	<0.001
SUD *n* in %	19.7		14		20.8		χ^2^(2) = 17.70	<0.001
Anxiety disorders *n* in %	43.6		42		20.8		χ^2^(2) = 17.70	<0.001
Clinical data								
Obsessive-compulsive inventory-revised	27.32	12.17	5.78	6.12	3.85	3.87	*F*_(2,294)_ = 261.82	<0.001
Beck depression inventory-II	18.81	10.70	3.46	4.38	2.53	3.32	*F*_(2,294)_ = 169.30	<0.001
State-trait-anxiety-inventory trait^a^	53.12	10.34	33.58	7.52	32.19	7.54	*F*_(2,288)_ = 191.21	<0.001
Harm avoidant motivation (OC-TCDQ)^a^	9.92	3.52	2.82	2.18	2.24	1.89	*F*_(2,288)_ = 271.80	<0.001
Sense of incompleteness (OC-TCDQ)^a^	15.20	5.40	5.08	3.79	4.39	3.27	*F*_(2,288)_ = 211.28	<0.001
Yale-brown obsessive-compulsive scale^b^	23.27	9.49						
Montgomery-Asberg depression rating scale^b^	13.23	9.24						
Age of onset^b^	23.27	9.49						
Performance data^c^								
Error rate in %	4.41	3.42	3.44	2.97	4.33	3.26	*F*_(2287)_ = 0.04	0.97
Reaction time for correct trials	436	48	462	54	438	48	*F*_(2287)_ = 0.18	0.84
Reaction time for errors	371	76	385	66	373	56		
Robust post-error slowing	40	45	52	60	39	47	*F*_(2287)_ = 0.28	0.76

s.d., standard deviation; OCD, obsessive-compulsive disorder; IQ, intelligence quotient; SUD, substance use disorders; OC-TCDQ, Obsessive-Compulsive Trait Core Dimensions Questionnaire.

a State-Trait-Anxiety-Inventory and OC-TCDQ scores were missing for six OCD patients.

b Yale-Brown Obsessive-Compulsive Scale, Montgomery-Asberg Depression Rating Scale and age of onset were only applicable in patients.

c For performance data, age was included as a covariate to account for group differences. Further, performance data of six participants were missing due to technical problems (*n* = 2 OCD patients, *n* = 2 relatives, and *n* = 2 healthy comparison participants).

OCD patients were recruited via the outpatient clinic at Humboldt-Universität zu Berlin, Germany, and fulfilled criteria for OCD as verified with the SCID. Exclusion criteria for patients were: a current or lifetime diagnosis of psychotic, SUD, or bipolar disorders as well as neuroleptic medication in the past 4 weeks and/or benzodiazepines use in the past 2 weeks. Fifty OCD patients were currently taking psychotropic medication (serotonin reuptake inhibitors: *n* = 38, serotonin–norepinephrine reuptake inhibitors: *n* = 6, tricyclic antidepressants: *n* = 6). The majority of patients had one to three comorbid axis-I disorders: major depression (*n* = 27 remitted, *n* = 31 current episode), dysthymia (*n* = 10), panic disorder (*n* = 3), agoraphobia (*n* = 2), social phobia (*n* = 10), specific phobia (*n* = 10), GAD (*n* = 5), unspecific somatoform disorder (*n* = 6), pain disorder (*n* = 1), hypochondria (*n* = 1), body dysmorphic disorder (*n* = 1), anorexia nervosa (*n* = 1), binge eating disorder (*n* = 2), tic disorder (*n* = 6), excoriation disorder (*n* = 1).

First-degree relatives of patients with OCD were recruited via OCD patients that were diagnosed and treated at the outpatient clinic at Humboldt-Universität zu Berlin. OCD patients gave written informed consent for contacting them. For all first-degree relatives of OCD patients the diagnosis of their affected relative was verified in a face-to-face SCID. OCD relatives were only included if they themselves were free of past or present OCD. Additional exclusion criteria applied to unaffected first-degree relatives of OCD patients were: lifetime diagnosis of psychotic, bipolar, or SUD, and psychotropic medication in the past 4 weeks.

Healthy comparison participants were recruited via public advertisements and were matched for age, gender, and education level to OCD patients. Exclusion criteria for comparison participants were as follows: psychoactive medication in the past 3 months; any current or past axis-I disorder; and a family history of OCD. The Family History Screen (Weissman *et al*., 2000) indicated that 28 healthy participants had first-degree relatives with depression, 27 first-degree relatives with anxiety disorders, and/or 27 reported to have first-degree relatives with SUD. Thus, the effects of a family history for depression, SUD (alcohol and substance use), and anxiety disorders (GAD, specific phobia, social phobia, agoraphobia, panic disorder) were examined. The number of cases did not allow to examine familial risk for other disorder such as schizophrenia, or attention-deficit/hyperactivity disorder or to separately analyze different anxiety disorders with sufficient power^2^. Note that family history information for unaffected first-degree relatives of individuals with SUD, anxiety, or depression was not verified by a direct interview with the family members but was derived via the Family History Screen (Weissman *et al*., 2000).

All participants gave written informed consent after receiving written and verbal information about the study, and were monetarily compensated for their time. The study was conducted in accordance with the ethical guidelines of the revised Declaration of Helsinki, as confirmed by the ethics committee of Humboldt-Universität zu Berlin.

### Measures

All participants completed the Beck-Depression-Inventory II (Beck *et al*., 1996), the Obsessive-Compulsive Inventory-Revised (Foa *et al*., 2002), and the State-Trait Anxiety Inventory (STAI, Spielberger *et al*., 1983) to assess self-reported severity of depressive, obsessive-compulsive, and anxiety symptoms, respectively. The Obsessive-Compulsive Trait Core Dimensions Questionnaire (Ecker and Gonner, 2008) was used to assess harm avoidance and sense of incompleteness in the participants. The Wortschatztest (Schmidt and Metzler, 1992) was applied to measure verbal intelligence. In addition, for patients only, severity of obsessive-compulsive and depressive symptoms was rated by trained clinicians using the Yale-Brown Obsessive-Compulsive Scale (Goodman *et al*., 1989) and the Montgomery-Asberg Depression Rating Scale (Montgomery and Asberg, 1979).

### Task

An arrow-version of the flanker task was administered using Presentation software (Neurobehavioral Systems, Inc., Albany, California). On each trial, five vertically aligned arrows were presented and participants were instructed to indicate the direction of the central arrow. The stimuli were presented for 100 ms, followed by a 1000 ms response interval, followed by an inter-trial interval that varied randomly between 200 and 1200 ms. Half of the trials were congruent and half were incongruent. Eight blocks with 60 trials and at the beginning 20 practice trials were presented. After each block, participants were reminded to respond both quickly and accurately. The duration of the experiment was about 25 min.

### Electroencephalographic recording and analyses

The electroencephalogram (EEG) was recorded from 61 Ag/AgCl-electrodes using an equidistant electrode montage (EASYCAP GmbH, Herrsching-Breitbrunn, Germany). External electrodes were placed below the eyes, below T1 (ground), and on the nasion. Channels were referenced to Cz during recording and impedances were below 5 kΩ. The EEG was sampled at a rate of 1000 Hz and a resolution of 0.1 *µ*V. EEG data were processed offline using BrainVision Analyzer 2.1 (Brain Products GmbH, Munich, Germany). The following filters were applied: a 50 Hz notch filter, a 30 Hz low-pass filter, and a 0.01 Hz high-pass filter. To correct for eye movements and eye blinks, an ocular correction independent component analysis was conducted. Data were re-referenced to average reference and response-locked segments were computed from 400 ms before and 1000 ms after response execution (baseline-correction −100 to 0 ms). Artifacts were excluded based on the following criteria: amplitude changes exceeding 50 *µ*V between consecutive data points, voltage differences of more than 200 *µ*V within a 200 ms interval, and voltage changes of less than 0.50 *µ*V within a 100 ms interval. Only participants with at least six artifact-free error trials were analyzed to ensure a reliable quantification of ERN (Olvet and Hajcak, 2009). ERN and CRN were quantified as the difference between the most negative peak occurring in a 150 ms post-response epoch and the immediately preceding positive peak at electrode FCz where error-related brain activity was maximal (Riesel *et al*., 2011). In addition, we also calculated ΔERN (ERN minus CRN) and quantified the ERN and CRN as the mean amplitude centered around the most negative peak occurring in a 150 ms epoch following the response at electrode FCz (peak ± 20 ms). Note that the pattern of results was replicated across the different ERN quantifications and related results are reported in online Supplementary information (SI 2). Results for the CRN are presented in the online Supplementary information (SI 1).

### Statistical analyses

One-way analyses of covariance were used to examine differences in ERN, symptom severity, and error rate between OCD patients, OCD relatives and healthy participants. Repeated-measurement analyses of covariance were used to analyze response times including *group* (OCD patients, OCD relatives, and healthy comparison participants) as between-subjects and *response type* (correct, error) as within-subjects factors. Age was included as a covariate in these analyses to control for age differences between groups. Additional analyses for the OCD group included *medication* (with *n* = 50, without medication *n* = 66) and *comorbidity* (with *n* = 86, without comorbidity *n* = 31) as between-subjects factors. We used hierarchical regressions to examine whether family history explained variance in ERN in unaffected comparison participants. Results for the CRN and the whole sample are presented in the online Supplementary information (SI 1 and SI 3). In a first step, the effects of age, depressive, and obsessive-compulsive symptoms were controlled. In a second step, family history information for SUD, depression, and anxiety was entered. In a third step, interactions between familial risk statuses were included. Regression and correlational analyses were performed to determine the relation between ERN and symptoms (online Supplementary information SI 4). Heritability estimates for error-related brain activity were computed using Sequential Oligogenic Linkage Analysis Routines (SOLAR) and are presented in the online Supplementary information (SI 5). All statistical tests were two-tailed with *α* = 0.05. Post-hoc comparisons were corrected using the Bonferroni procedure. Statistical analyses were conducted with SPSS (Version 21.0, Chicago, USA).

## Results

### Demographic and behavioral data

Table 1 shows demographic, clinical, behavioral measures, and the statistics for the comparison between groups. Patients with OCD and healthy comparison participants did not differ in age (*t*_245_ = 0.14, *p* = 0.89). OCD relatives were significantly older compared to patients (*t*_165_ = 6.82, *p* < 0.001) and healthy comparison participants (*t*_178_ = 6.71, *p* < 0.001). Groups did not differ in gender and verbal intelligence quotient (IQ). OCD patients scored higher on symptom measures than OCD relatives (depressive symptoms: *t*_165_ = 9.78, *p* < 0.001; obsessive-compulsive symptoms: *t*_165_ = 11.87, *p* < 0.001; anxiety symptoms: *t*_159_ = 12.00, *p* < 0.001; harm avoidance: *t*_159_ = 13.16, *p* < 0.001; sense of incompleteness: *t*_159_ = 11.98, *p* < 0.001) and healthy comparison participants (depressive symptoms: *t*_245_ = 16.47, *p* < 0.001; obsessive-compulsive symptoms: *t*_245_ = 20.85, *p* < 0.001; anxiety symptoms: *t*_239_ = 18.12, *p* < 0.001; harm avoidance: *t*_239_ = 21.48, *p* < 0.001; sense of incompleteness: *t*_239_ = 19.10, *p* < 0.001). OCD relatives did not differ from healthy comparison participants in depressive (*t*_178_ = 1.52, *p* = 0.13), anxiety symptoms (*t*_178_ = 1.11, *p* = 0.27), harm avoidance (*t*_178_ = 1.74, *p* = 0.08) and sense of incompleteness (*t*_178_ = 1.21, *p* = 0.23). However, unaffected first-degree relatives reported slightly more obsessive-compulsive symptoms (*t*_178_ = 2.51, *p* = 0.01).

Groups did not differ in error rates. Correct responses were slower compared to errors (*F*_1,287_ = 28.31, *p* < 0.001, *η*^2^_P_ = 0.09). A main effect of the covariate *age* (*F*_1,287_ = 52.47, *p* < 0.001, *η*^2^_P_ = 0.16) reflected that increasing age was associated with slower responses (*r* = 0.49, *p* < 0.001). No main effect of *group* was observed after accounting for age differences. Robust post-error slowing (difference in reaction time between post-error and the associated pre-error trial) did not differ between groups.

### Error-related negativity

**Fig. 1.**
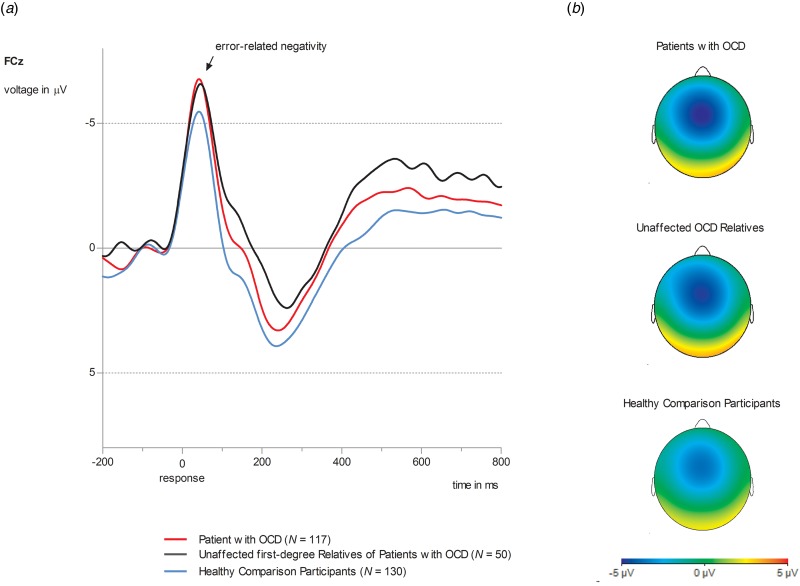
(*a*) Grand average waveforms at electrode site FCz for ERN amplitude in OCD patients (red lines, *N* = 117), unaffected first-degree relatives of OCD patients (black lines, *N* = 50) and healthy comparison participants (blue lines, *N* = 130). (*b*) Topographies of error-related brain activity for OCD patients, OCD relatives and healthy comparison participants depicting the mean activity in the time window from 0 to 100 ms after response execution.

Figure 1 displays ERN for OCD patients, unaffected first-degree relatives of OCD patients and unaffected comparison participants. Results for the CRN are presented in the online Supplementary information (SI 1). A significant main effect of *group* (*F*_2,293_ = 4.39, *p* = 0.005, *η*^2^_P_ = 0.04, *d* = 0.39) was observed. Both OCD patients and unaffected relatives of OCD patients showed enhanced ERN amplitudes compared to unaffected comparison participants [OCD patients *v.* healthy comparison participants: *mean difference* = 1.39, s.e. = 0.59, *p* = 0.05, 95% confidence interval (CI) (0.04–2.80); OCD relatives *v.* healthy comparison participants: *mean difference* = 2.49, s.e. = 0.83, *p* = 0.009, 95% CI (0.48–4.50)]. OCD patients and OCD relatives did not differ in ERN [*mean difference* = 0.19, s.e. = 0.81, *p* = 0.81, 95% CI (−1.40 to 1.79)]. A significant main effect of the covariate *age* (*F*_1,293_ = 7.85, *p* = 0.005, *η*^2^_P_ = 0.03) was observed. Age and ERN showed a positive correlation (*r* = 0.12; *p* = 0.05) with increasing age being associated with more positive (i.e. smaller) amplitudes. For patients, neither a main effect for *medication* (*F*_1,114_ = 0.02, *p* = 0.89, *η*^2^_P_ = 0.00), or *comorbidity* (*F*_1,114_ = 0.44, *p* = 0.51, *η*^2^_P_ = 0.004) nor significant interactions involving these variables were observed (*p* values >0.33). When analyzing associations with symptoms across the whole sample, only harm avoidance showed a correlation with ERN (*r* = −0.13, *p* = 0.03). Higher harm avoidant motivation was associated with more negative, i.e. larger amplitudes. No other associations with symptoms or verbal IQ in the whole sample as well as symptom dimensions and clinical features in OCD patients were observed (see online Supplementary information SI 4).

**Fig. 2.**
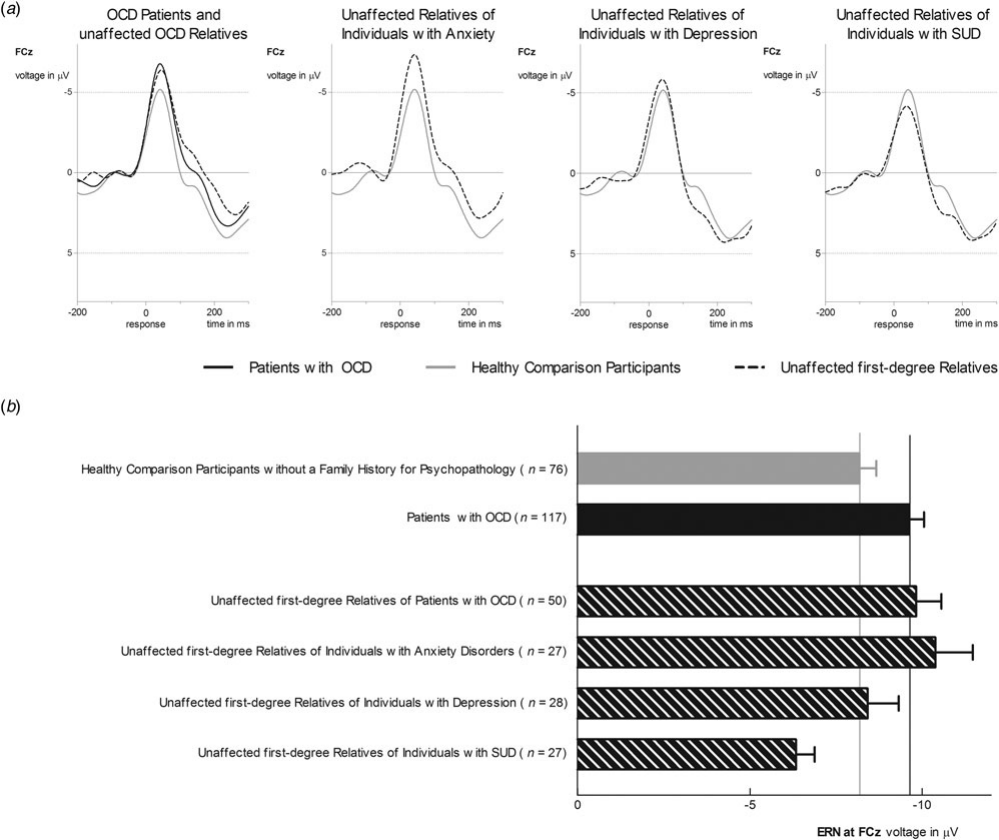
(*a*) Grand average waveforms at electrode site FCz for ERN amplitude in healthy comparison participants without a family history for a major psychiatric disorder (gray line) compared to unaffected first-degree relatives of patients with OCD and OCD patients (first column), unaffected first-degree relatives of individuals with anxiety disorders (second column), unaffected first-degree relatives of individuals with depression (third column), and unaffected first-degree relatives of individuals with SUD (last column). (*b*) Bar chart depicting ERN amplitudes for healthy comparison participants, OCD patients, and unaffected first-degree relatives. Values are means, with error bars depicting standard errors.

Using hierarchical regressions, we examined whether family history information explained variance in ERN in healthy comparison participants after adjusting for age, gender and symptom severity (Table 2, Fig. 2). Familial risk for SUD and anxiety (i.e. having a first-degree relative with this disorder) significantly predicted ERN magnitude in healthy participants. Unaffected first-degree relatives of individuals with SUD (i.e. healthy participants at familial risk for SUD) reported lower ERN amplitudes compared to those without familial risk for SUD [*t*_128_ = 2.49, *p* = 0.01, *d* = 0.54, *mean difference* = 2.40, s.e. = 0.96, 95% CI (0.49–4.30)]. Further, increased ERN amplitudes were observed in unaffected first-degree relatives of individuals with anxiety [i.e. healthy participants with a family history for anxiety; *t*_128_ = 2.83, *p* = 0.005, *d* = 0.61, *mean difference* = 2.70, s.e. = 0.96, 95% CI (0.81–4.59)]. Unaffected first-degree relatives of individuals with anxiety disorder (*M* = −10.37, s.d. = 5.66) did not differ in ERN from OCD patients (*M* = −9.62, s.d. = 4.58) and OCD relatives (*M* = −9.82, s.d. = 5.22; *F*_2,190_ = 0.72, *p* = 0.49, *η*^2^_P_ = 0.008). Entering interactions between risk statuses does not significantly enhance the model fit (change in *F* = 0.75) and no significant interactions were observed.

**Table 2.** Regression model for the ERN to examine effects of family history on error processing in unaffected comparison participants

Variables	*R*^2^	*F*	df1, df2	*p-*value	*B*	s.e.	*β*	*t*-value	*p* value
DV: ERN	0.03	0.70	5, 124	0.62					
Age					0.06	0.04	−0.03	−0.35	0.11
Gender					−0.29	0.83	0.14	1.60	0.73
Obsessive-compulsive inventory-R					−0.08	0.11	−0.07	−0.75	0.46
Beck depression inventory-II					−0.01	0.16	0.009	−0.08	0.74
State-trait-anxiety-inventory trait					0.06	0.07	0.10	0.77	0.44
DV: ERN	0.13	2.29	8, 121	**0.03**					
Age					0.06	0.04	0.12	1.40	0.16
Gender					−0.27	0.80	−0.03	−0.34	0.74
Obsessive-compulsive inventory-R					−0.06	0.11	−0.05	−0.56	0.58
Beck depression inventory-II					−0.01	0.16	−0.01	−0.09	0.93
State-trait-anxiety-inventory *trait*					0.05	0.07	0.07	0.65	0.52
Risk for SUD					2.37	1.01	0.21	2.35	**0.02**
Risk for anxiety					−2.94	1.06	−0.26	−2.77	**0.007**
Risk for depression					0.57	1.10	0.05	0.52	0.61
DV: ERN	0.13	1.86	11, 118	**0.05**					
Age					0.06	0.04	0.13	1.48	0.14
Gender					−0.27	0.81	−0.03	−0.33	0.75
Obsessive-compulsive inventory-R					−0.07	0.11	−0.06	−0.66	0.51
Beck depression inventory-II					−0.05	0.17	−0.04	−0.29	0.78
State-trait-anxiety-inventory trait					0.06	0.07	0.10	0.80	0.43
Risk for SUD					2.77	1.32	0.25	2.11	**0.04**
Risk for anxiety					−3.09	1.38	−0.33	−2.67	**0.009**
Risk for depression					1.41	1.70	0.13	0.83	0.41
Interaction risk for SUD × risk for anxiety					2.97	2.78	0.15	1.07	0.29
Interaction risk for SUD × risk for depression					−3.09	2.50	−0.20	−1.23	0.22
Interaction risk for anxiety × risk for depression					0.29	2.43	0.02	0.12	0.91

DV, dependent variable; SUD, substance use disorders.

Significant values are printed in bold.

## Discussion

Neural error-signals were examined as an endophenotype for OCD by comparing it between patients with OCD, unaffected first-degree relatives of OCD patients and healthy participants without a family history for OCD. Furthermore, we evaluated whether neural error-signals may represent a transdiagnostic endophenotype by analyzing unaffected first-degree relatives of individuals with SUD, depression, and anxiety disorders. Three main findings were observed: first, both patients with OCD and unaffected first-degree relatives of OCD patients showed increased ERN amplitudes compared to healthy comparison participants. Second, the increase in ERN magnitude was not specific to OCD as unaffected first-degree relatives of individuals with anxiety disorders were also characterized by increased ERN amplitudes compared to healthy comparison participants. Third, unaffected first-degree relatives of individuals with SUD showed reduced ERN amplitudes compared to healthy comparison participants, OCD patients, and relatives of individuals with OCD or anxiety. Overall, these results support the notion that neural error-signals represent a potential endophenotype not only for OCD but across different psychiatric disorders.

A useful endophenotype is a robust, state-independent quantitative deviation that is found in patients and unaffected family members. It is heritable, easy and reliable to assess and informative about the pathophysiology of the disorder (Gottesman and Gould, 2003; Miller and Rockstroh, 2013). Enhanced neural error-signals in OCD fulfill most of these criteria. The ERN has been shown to have good psychometric properties including validity and reliability (Olvet and Hajcak, 2009; Weinberg and Hajcak, 2011; Riesel *et al*., 2013). Increased neural error-signals are robustly associated with OCD (Endrass and Ullsperger, 2014; Gillan *et al*., 2017) and can also be found in unaffected first-degree relatives of OCD patients (Riesel *et al*., 2011; Carrasco *et al*., 2013). Further, elevated ERN amplitudes in OCD persist after symptom reduction due to CBT (Hajcak *et al*., 2008; Huyser *et al*., 2011; Riesel *et al*., 2015). A twin study suggested a heritability of about 50% for ERN (Anokhin *et al*., 2008) and we derived comparable heritability estimates ranging between 22% and 48% (online Supplementary information SI 5). Altogether, these results highlight that increased neural error-signals represent a promising endophenotype for OCD.

The current study indicates that increased ERN amplitudes are also found in healthy participants at familial risk for anxiety (i.e. unaffected first-degree relatives of individuals with anxiety disorders). This is consistent with studies reporting increased ERN amplitudes for several anxiety disorders including GAD, social anxiety, and health anxiety (Manoach and Agam, 2013; Gillan *et al*., 2017; Riesel *et al*., 2017). These disorders are frequently comorbid and share symptoms that have been linked to elevated neural error signals such as repetitive behavior (Manoach and Agam, 2013), worry (Moser *et al*., 2013), and anxiety-proneness (Cavanagh and Shackman, 2014) pointing to shared pathophysiological mechanisms. The observed increase in ERN in unaffected relatives with a family history for anxiety disorders or OCD compared to healthy controls provides important evidence for the assumption that increased neural error-signals represent a transdiagnostic endophenotype indicating a shared vulnerability for OCD and anxiety. This is further supported by results indicating the state-independence of increased ERN amplitudes not only in OCD (Hajcak *et al*., 2008; Huyser *et al*., 2011; Riesel *et al*., 2015) but also in social anxiety disorder (Kujawa *et al*., 2016). Finally, increased ERN amplitudes prospectively predict the development of anxiety symptoms (Lahat *et al*., 2014; Lamm *et al*., 2014; Meyer *et al*., 2015; Meyer *et al*., 2018) highlighting the predictive validity of neural error signals.

The current study did not find support for altered ERN amplitudes in unaffected first-degree relatives of individuals with depression (i.e. healthy individuals at familial risk for depression). Results regarding increased ERN amplitudes in depression are inconsistent (Gillan *et al*., 2017), pointing toward increases (e.g. Chiu and Deldin, 2007), as well as decreases (e.g. Weinberg *et al*., 2016), or no differences (e.g. Schoenberg, 2014). Overall, neural error-signals in depression seem to be more affected by symptom state and severity (Schrijvers *et al*., 2009), as well as subtype (Weinberg *et al*., 2016). An increase was reported for mild to moderate depression, but not in severely depressed patients with melancholic or anhedonic features that even showed a blunted response to errors (Schrijvers *et al*., 2009; Weinberg *et al*., 2016). Further, alterations in error-monitoring disappear after symptom reduction (Schrijvers *et al*., 2009). Together with our results showing normal error-monitoring in individuals at familial risk for depression this suggests that ERN alterations seem not to be an endophenotypic trait marker of depression. Observed alterations may rather reflect a state marker or a result from overlap/comorbidity with relevant transdiagnostic phenotypes. This supports some specificity and suggests that increased neural error-signals may distinguish vulnerability for OCD and anxiety from depression.

Finally, unaffected first-degree relatives of individuals with SUD showed reduced error-related brain activity compared to healthy participants without a family history for SUD. This supports that a blunted neural response to errors may not only be found in patients with SUD (Luijten *et al*., 2014; Gillan *et al*., 2017) but can also be seen in individuals at increased risk for SUD. This confirms a previous study in adolescents at high-risk for SUD that were characterized by diminished neural error-signals (Euser *et al*., 2013). Further, the suitability of reduced error-related brain activity as an endophenotype for SUD is supported by longitudinal studies showing that it predicts relapse (Luo *et al*., 2013; Marhe *et al*., 2013) and the initiation of tobacco use (Anokhin and Golosheykin, 2015). Besides SUD, several studies suggest that reduced ERN amplitudes are also found in schizophrenia (Martin *et al*., 2018), bipolar disorder (Minzenberg *et al*., 2014), and inconsistently in autism and attention-deficit/hyperactivity disorder (Manoach and Agam, 2013; Luijten *et al*., 2014; Gillan *et al*., 2017). This suggests that a blunted neural response to errors is common to different disorders and may reflect a shared pathophysiological mechanism.

In summary, the present results support the utility of neural error-signals as a dimensional, transdiagnostic endophenotype with an enhancement in ERN reflecting vulnerability for OCD and anxiety, and a reduction indicating risk for SUD. OCD and anxiety disorders are frequently comorbid (Ruscio *et al*., 2010) and share prominent clinical features such as heightened harm avoidance (Kampman *et al*., 2014), worry (Yook *et al*., 2010; Dar *et al*., 2015), increased intolerance of uncertainty (Tolin *et al*., 2003; Holaway *et al*., 2006), as well as reassurance seeking and checking behavior (Kobori and Salkovskis, 2013; Weinberg *et al*., 2015*b*; Halldorsson and Salkovskis, 2017). These symptoms have also been related to hyperactive neural error signals that are observed across OCD and anxiety disorders (e.g. Moser *et al*., 2013; Cavanagh and Shackman, 2014; Weinberg *et al*., 2015*b*). Collectively, similarities in symptoms, neural substrate, and comorbidity point to a partly shared etiology involving alterations in error-monitoring. More specifically, differences in neural error signals that are generated by the ACC (Debener *et al*., 2005; Manoach and Agam, 2013; Grutzmann *et al*., 2016), are assumed to trigger adaptations in cognition, affect, and control implementation to avoid future negative outcomes (Shackman *et al*., 2011; Cavanagh and Shackman, 2014). In this regard, hyperactive neural error-signals (i.e. increased sensitivity to errors) in OCD and anxiety may reflect over-controlled responses and an error- and harm-avoidant response style. Moreover, elevated neural error-signals are not only found in patients with these disorders but seem to indicate vulnerability for OCD and anxiety that persists independent of symptom status and are present in individuals at risk. On the other hand, reduced neural error-signals may indicate under-controlled behavior (i.e. reduced responsiveness to errors/negative consequences and deficits in adaptive control) and seem to be related to risk for substance use. Overall, our results add to numerous findings pointing to common etiological factors shared between different psychiatric disorders which encompass structural abnormalities in ACC and disruptions in cognitive control (Goodkind *et al*., 2015; McTeague *et al*., 2017). Error-monitoring dysfunctions fit nicely into this picture since they have been linked to cognitive control (Cavanagh and Shackman, 2014) and are assumed to depend on activity in the ACC (Debener *et al*., 2005; Manoach and Agam, 2013). The ACC is implicated in the processing and integration of negative affect, cognitive conflict, and pain, as well as the implementation of adaptive control (Shackman *et al*., 2011) and its function seems to be critically involved in mental health.

Some limitations have to be noted. Groups differ in age, however, results are corrected for age differences. Some patients were medicated and some had current comorbid disorders. But in line with previous reports, our findings were not affected by medication or comorbidity in patients (Stern *et al*., 2010; Riesel *et al*., 2015). Family history methods have high specificity (above 0.9), but rather low sensitivity and false negative diagnoses are common (Rougemont-Buecking *et al*., 2008; Vandeleur *et al*., 2008; Vandeleur *et al*., 2015). This may have led to an underestimation of family history for depression, SUD, and anxiety. Further, in the current study diagnostic confidence differs between unaffected first-degree relatives of OCD patients and unaffected first-degree relatives of individuals with disorders other than OCD. All unaffected first-degree relatives of patients with OCD were recruited via patients of the outpatient clinic for OCD at Humboldt-Universität zu Berlin, where they were carefully diagnosed using the SCID. Thus, the OCD diagnosis of the affected first-degree relative was verified by a direct SCID. Family history information for disorders other than OCD was assessed with a family history interview (Weissman *et al*., 2000). Due to restrictions by data protection regulations, diagnostic information was not verified with a direct interview of the affected family member. Further, prevalence rates were too small to separately analyze the effects of specific anxiety disorders. Finally, the endophenotype concept is not without criticism (Kendler and Neale, 2010; Miller and Rockstroh, 2013; Iacono *et al*., 2017). The success of the endophenotype strategy to identify predisposing genes has been demonstrated in non-psychiatric disorders, such as cardiac syndromes (Keating *et al*., 1991; Vincent *et al*., 1992; Keating and Sanguinetti, 2001), but remains limited for psychiatric disorders (Iacono *et al*., 2017). The genetic basis of endophenotypes is complex (Iacono *et al*., 2017). For the ERN results regarding its genetic foundation are inconsistent, rely on rather small samples, and replications are needed (Manoach and Agam, 2013). Furthermore, as Kendler and Neale (2010) have noted, endophenotypes can either causally mediate between genes and the clinical phenotype, or be risk-indicators that share genes with the phenotype. Nevertheless, even complex endophenotypes are presumably simpler than the complex disorder. Besides pointing out genetic influences, endophenotypes can help to illuminate disorder mechanisms and familial environmental risk factors (Kendler and Neale, 2010) and potentially provide targets for interventions. Further, endophenotypes and especially profiles of different potential endophenotypes have the power to differentiate disorders that overlap symptomatically and in pathophysiological mechanisms, as has been shown for psychotic disorders including schizophrenia, bipolar, and schizoaffective disorder (e.g. Benes, 2007; Braff and Tamminga, 2017; Ivleva *et al*., 2017). Thus, they may inform new ways of categorizing disorders (Insel *et al*., 2010) and can foster individualized and precision medicine (Insel and Cuthbert, 2015). Finally, the clinical relevance of neural error-signals as a risk indicator or endophenotype is further supported by studies demonstrating the predictive validity for the onset of disorders or symptoms (Lahat *et al*., 2014; Lamm *et al*., 2014; Anokhin and Golosheykin, 2015; Meyer *et al*., 2015, 2018; Kessel *et al*., 2016), as well as treatment outcome (Luo *et al*., 2013; Marhe *et al*., 2013) across different mental disorders.

The current study is the first to examine error-monitoring in unaffected individuals with familial risk for several disorders simultaneously. A diagnosis of OCD, familial risk for OCD or anxiety disorders were associated with increased error-related brain activity compared to healthy control participants without a family history for psychopathology. In contrast, familial risk for SUD was associated with a reduced neural response to errors compared to the healthy control group as well as patients with OCD and relatives with a family history of OCD or anxiety. The results provide important evidence for variations in neural error-signals being a promising transdiagnostic endophenotype reflecting vulnerability for the development of OCD, anxiety, or SUD. Further, they add to results suggesting that structural ACC alterations and deficits in cognitive control represent a shared neurobiological substrate across mental disorders.

## Footnotes

† The notes appear after the main text.

## Notes

1 A smaller negative component, the correct-related negativity (CRN, Ford, 1999), can be seen following correct responses. Similarities in topography, timing, and source suggest that both components reflect similar and overlapping neuronal and cognitive processes (Vidal *et al.*, 2000; Hoffmann & Falkenstein, 2010; Klawohn *et al.*, 2014). Overall, anxiety and OCD have more closely and consistently been related to hyperactive error-monitoring. The primary focus of the present paper is on the ERN and related CRN results are presented in the online Supplementary information (SI 1).

2 Number of healthy control participants with a family history for other disorder: schizophrenia (*n* = 1), bipolar disorder (*n* = 3), attention-deficit/hyperactivity disorder (*n* = 7), anorexia nervosa (*n* = 3), tic disorder (*n* = 1), excoriation disorder (*n* = 2), and body dysmorphic disorder (*n* = 3).

## Supplementary material

For supplementary material accompanying this paper visit https://doi.org/10.1017/S0033291719000199. click here to view supplementary material
