# Supplementary material for: Error-related brain activity as a transdiagnostic endophenotype for obsessive-compulsive disorder, anxiety and substance use disorder
Source: Psychol Med. 2019 Feb 12;49(7):1207–17. doi: 10.1017/S0033291719000199 (PMC6498788; doi:10.1017/S0033291719000199)
Supplement: Supplementary file 1 [file S0033291719000199sup001.docx]

**Supplemental Information**

Content of supplemental information (SI):

1. Supplementary Figure 1: Participant flowchart
2. SI 1: Results for the correct-related negativity (CRN)
3. SI 2: Results for different quantifications of the ERN
4. SI 3: Hierarchical regression analysis examining the effects of familial risk for psychopathology on the ERN in the whole sample.
5. SI 4: Associations between ERN and symptom measures as well as obsessive-compulsive symptom severity and symptom dimensions.
6. SI 5: Heritability analysis for neural error-signals.

**Supplementary Figure 1: Participant flowchart**


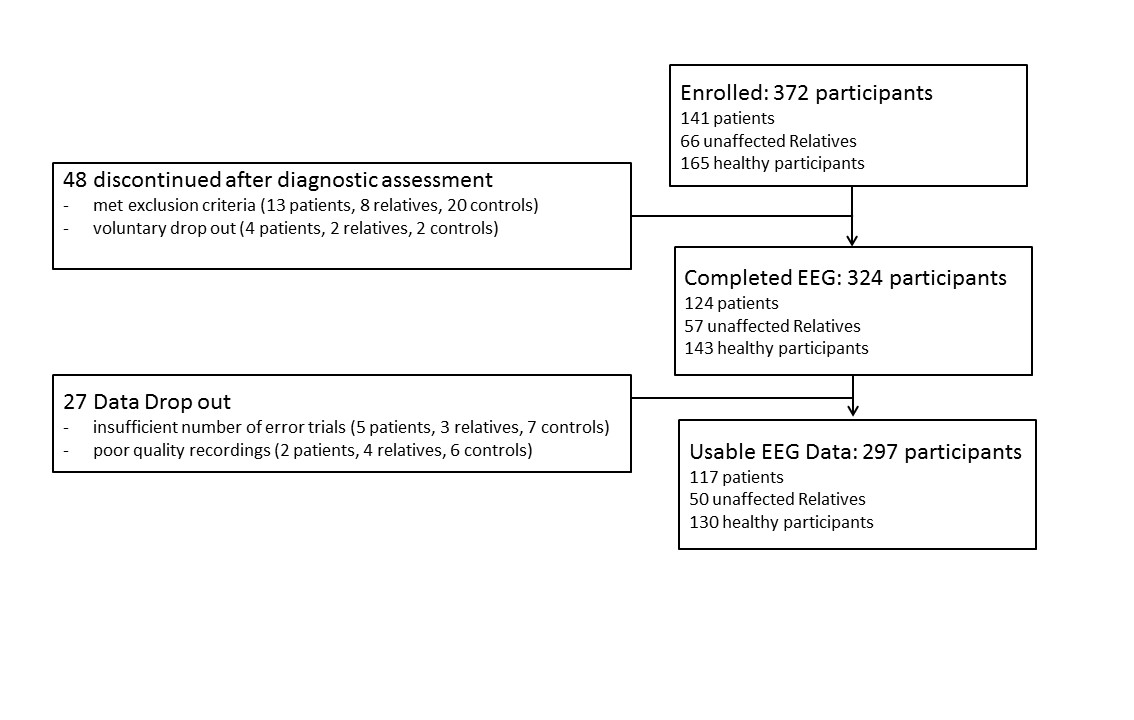
***SI 1: Results for the correct-related negativity.***

One-way analyses of covariance were used to examine differences in CRN between OCD patients, OCD relatives and healthy participants. CRN did not differ between groups (F_2,293_=0.18, p=.84, *η^2^_P=_*.001, see supplementary Figure 2). A significant effect of the covariate *age* was found (F_1,293_=6.38, p=.01, *η^2^_P=_*.02). Age showed a negative correlation with the CRN (*r*=-.16, *p*=.004) with increasing age being associated with more negative (i.e., larger) amplitudes. Furthermore, a hierarchical regression analysis was performed to examine whether risk for psychopathology explained variance in CRN in healthy comparison participants (Table S1). No significant effects of family history for SUD, depressive disorders or anxiety on CRN have been observed. Age and gender significantly influences CRN amplitude. Participants with older age and female participants had larger (i.e., more negative) ERN amplitudes.


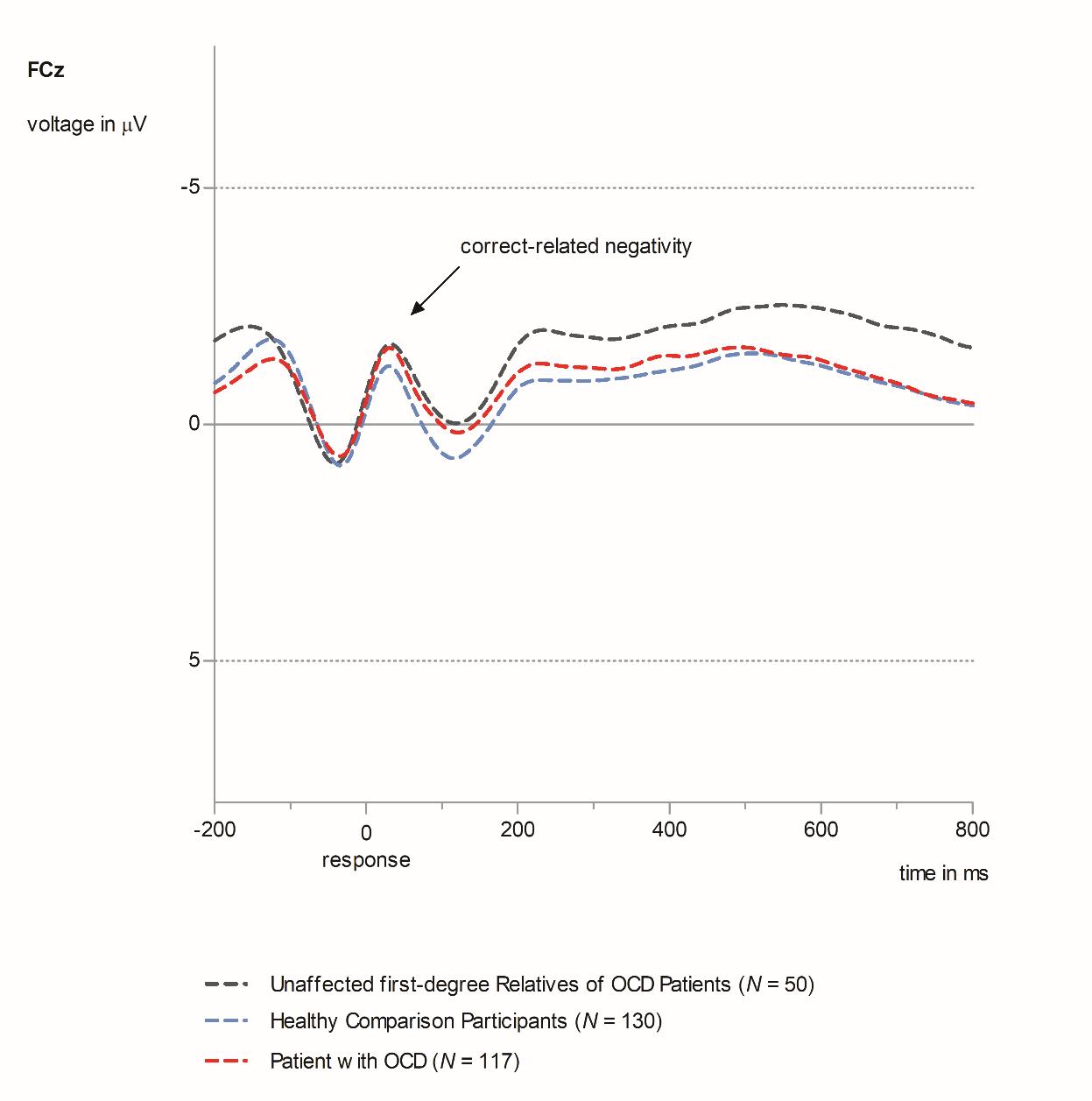
Supplementary Figure 2: Grand average waveforms at electrode site FCz for correct-related negativity amplitude in OCD patients (red lines, *N* = 117), unaffected first-degree relatives of OCD patients (black lines, *N* = 50) and healthy comparison participants (blue lines, N = 130).

**Supplementary Table 1:** Regression model to examine effects of family history on correct-related negativity amplitude in unaffected comparison participant

| Variables | R² | *F* | df1, df2 | *p-*value | B | SE | *β* | *t*-value | *p*-value |
| --- | --- | --- | --- | --- | --- | --- | --- | --- | --- |
| DV: CRN  Age  Gender  Obsessive-Compulsive Inventory-R  Beck Depression Inventory-II  State-Trait-Anxiety-Inventory *trait* | 0.12 | 3.49 | 5, 124 | .006 | -0.04  -1.02  0.02  0.02  -0.04 | 0.02  0.34  0.05  0.06  0.03 | -.19  -.26  .04  .03  -.16 | -2.23  -3.04  0.41  0.24  -1.40 | .**03**  **.003**  .68  .81  .16 |
| DV: CRN  Age  Gender  Obsessive-Compulsive Inventory-R  Beck Depression Inventory-II  State-Trait-Anxiety-Inventory *trait*  Risk for SUD  Risk for Anxiety  Risk for Depression | 0.13 | 7.66 | 8, 121 | .03 | -0.04  -1.01  0.02  0.02  -0.04  0.23  -0.22  -0.10 | 0.02  0.34  0.05  0.07  0.03  0.43  0.45  0.47 | -.19  -.26  .04  .04  -.17  .05  -.05  -.02 | -2.72  -2.97  0.47  0.29  -1.44  0.53  -0.49  -0.22 | **.03**  **.004**  .64  .77  .15  .60  .63  .83 |
| DV: CRN  Age  Gender  Obsessive-Compulsive Inventory-R  Beck Depression Inventory-II  State-Trait-Anxiety-Inventory *trait*  Risk for SUD  Risk for Anxiety  Risk for Depression  Interaction Risk for SUD x Anxiety  Interaction Risk for SUD x Depression  Interaction Risk for Anxiety x Depression | 0.15 | 1.90 | 11, 118 | .05 | -0.04  -1.07  0.03  0.01  -0.04  0.72  0.08  0.77  0.26  -1.55  -0.88 | 0.02  0.34  0.05  0.07  0.03  0.56  0.59  0.72  1.18  1.06  1.03 | -.19  -.28  .06  -.01  -.15  .15  -.02  -.16  .03  -.23  -.15 | -2.16  -3.10  0.62  -0.07  -1.21  1.29  0.14  1.07  0.22  -1.47  -0.86 | **.03**  **.002**  .54  .95  .23  .20  .89  .29  .83  .15  .39 |

Note: DV, dependent variable; CRN correct-related negativity; OCI-R, Obsessive-Compulsive Inventory-Revised; BDI-II, Beck Depression Inventory-II; SUD, Substance Use Disorder; OCD, obsessive–compulsive disorder

***SI 2: Results for different quantifications of the ERN***

ERN and CRN were quantified as the difference between the most negative peak occurring in a 150ms post-response epoch and the immediately preceding positive peak at electrode FCz where error-related brain activity was maximal. Further, we calculated ΔERN (ERN minus CRN). In addition, we also quantified the ERN and CRN as mean amplitude centered around the most negative peak occurring in a 150ms epoch following the response at electrode FCz (peak ± 20ms). One-way analyses of covariance were used to examine differences in ERN between OCD patients, OCD relatives and healthy participants while accounting for group differences in age. The main effect of group was found for all ERN quantifications (ERN peak-to-peak: F_2,293_=4.39, p=.005, *η^2^_P=_*.04, d=0.39; ERN area around peak: F_2,293_=3.79, p=.02, *η^2^_P=_*.03, d=0.32; ΔERN: F_2,293_=5.44, p=.005, *η^2^_P=_*.04, d=0.39). Similarly, the effect of the covariate age, was also observable across the different quantification methods (ERN peak-to-peak: F_1,293_=7.85, p=.005, *η^2^_P=_*.03; ERN area around peak: F_1,293_=9.78, p=.002, *η^2^_P=_*.03; ΔERN: F_1,293_=16.12, p<.001, *η^2^_P=_*.05).

Hierarchical regression analyses were used to examine the effect of family history on ERN in healthy comparison participants after accounting for the effects of age, gender and symptom severity. Regardless of quantification method we observed an effect of family history for anxiety (ERN peak-to-peak: *β* = -.26, *t* = -2.77, *p* = .007, *η^2^_P_* = .03; ERN area around peak: *β* = -.22, *t* = -2.27, *p* = .03; ΔERN: *β* = -.26, *t* = -2.80, *p* = .006), and SUD (ERN peak-to-peak: *β* = .21, *t* = 2.35, *p* = .02, ERN area around peak: *β* = .18, *t* = 1.96, *p* = .05; ΔERN: *β* = .20, *t* = 2.32, *p* = .02). Entering interactions between risk statuses did not significantly enhance the model fit and no significant interactions were observed.

***SI 3: Hierarchical regression analyses for error-related negativity amplitude in the whole sample***

A hierarchical regression analyses were performed to examine whether risk for psychopathology explained variance in ERN amplitude in the whole sample including OCD patients (Table S2 and S3). Again, age and symptom-severity scores were entered in a first step. In a second step, family history information for OCD, SUD, depression and anxiety were entered. In a third step, we entered interactions between familial risk statuses. Because participants were nested within families, we used robust standards errors (Huber-White sandwich estimator) to account for shared environmental and genetic effects and non-independence of residuals in the regression analyses using the whole sample (Lee *et al.*, 2013). The regression analyses indicated that besides the effect of OCD, familial risk for SUD significantly predicted ERN amplitude (Table S2 and S3). Participants at risk for SUD reported lower ERN amplitudes across the whole sample (ERN peak-to-peak: t_295_=2.73, p<.01, d=0.32; ERN area around peak: t_295_=2.53, p<.05, d=0.37). No interactions between OCD and SUD were obtained reflecting that effects on ERN amplitude were independent and additive. A significant interaction between OCD and anxiety was found. Post-hoc tests indicated that risk for anxiety modulates ERN amplitude in unaffected comparison participants (i.e., individuals without OCD or familial risk for OCD, ERN peak-to-peak: t_128_=1.93, p=.05; ERN area around peak: t_128_=2.15, p<.05, d=0.46) whereas risk for anxiety does not change ERN in OCD patients (ERN peak-to-peak: t_115_=1.14, p=.26; ERN area around peak: t_115_=0.22, p=.83) and OCD relatives (ERN peak-to-peak: t_48_=1.13, p=.26; ERN area around peak: t_48_=1.56, p=.13). Unaffected participants with familial risk for anxiety disorders did not differ in ERN from OCD patients and OCD relatives (ERN peak-to-peak: F_2,193_=0.03, p=.97, *η^2^_P=_*.00; ERN area around peak: F_2,193_=0.09, p=.91, *η^2^_P=_*.00).

**Supplementary Table 2**: Regression model for the ERN (peak-to-peak) to examine effects of family history on error-processing in the whole sample

| Variables | R² | *F* | df1, df2 | *p-*value | B | Robust SE | *β* | *t*-value | *p*-value |
| --- | --- | --- | --- | --- | --- | --- | --- | --- | --- |
| DV: ERN  Age  OCI-R  BDI-II | 0.01 | 1.28 | 3, 291 | .28 | 0.04  -0.02  0.01 | 0.02  0.03  0.04 | .10  -.04  .003 | 2.67  -0.75  0.22 | .09  .46  .84 |
| DV: ERN  Age  OCI-R  BDI-II  Risk for SUD  Risk for Anxiety  Risk for Depression  Risk for OCD | 0.07 | 3.84 | 7, 289 | <.001 | 0.06  0.02  0.02  1.55  -0.08  -0.01  -2.35 | 0.02  0.03  0.04  0.71  0.63  0.61  0.94 | .15  .07  .06  .13  -.01  -.001  -.25 | 2.48  0.72  0.69  2.18  -0.13  -0.01  -2.49 | **.01**  .47  .49  **.03**  .89  .99  **.01** |
| DV: ERN  Age  OCI-R  BDI-II  Risk for SUD  Risk for Anxiety  Risk for Depression  Risk for OCD  OCD x Depression  OCD x SUD  OCD x Anxiety  SUD x Anxiety  SUD x Depression  Anxiety x Depression | 0.11 | 3.48 | 13, 283 | <.01 | 0.06  0.03  0.02  1.51  -3.38  -0.07  -2.49  -1.12  -1.99  3.24  1.22  0.85  1.90 | 0.02  0.03  0.04  1.04  1.25  0.82  0.94  1.10  1.69  1.23  1.65  1.52  1.19 | .16  .08  .05  .13  -.34  -.01  -.27  -.11  -.13  .30  .07  .06  .16 | 2.61  0.89  0.60  1.45  -2.71  -0.08  -2.64  -1.01  -1.18  2.65  0.7  0.56  1.59 | **.01**  .37  .55  .15  **.007**  .93  **.008**  **.**31  .24  **.008**  .46  .57  .11 |

Note: DV, dependent variable; ERN, error-related negativity; OCI-R, Obsessive-Compulsive Inventory-Revised; BDI-II, Beck Depression Inventory-II; SUD, Substance Use Disorder; OCD, obsessive–compulsive disorder;

**Supplementary Table 3**: Regression model for the ERN (area around peak) to examine effects of family history on error-processing in the whole sample

| Variables | R² | *F* | df1, df2 | *p-*value | B | Robust SE | *β* | *t*-value | *p*-value |
| --- | --- | --- | --- | --- | --- | --- | --- | --- | --- |
| DV: ERN  Age  OCI-R  BDI-II | 0.03 | 2.89 | 3, 293 | <.05 | 0.05  -0.02  0.01 | 0.02  0.03  0.03 | .15  -.08  .03 | 2.69  -0.91  0.32 | **.007**  .36  .75 |
| DV: ERN  Age  OCI-R  BDI-II  Risk for SUD  Risk for Anxiety  Risk for Depression  Risk for OCD | 0.07 | 3.12 | 7, 289 | <.01 | 0.06  -0.01  0.03  1.39  -0.31  -0.30  -1.37 | 0.02  0.03  0.03  0.67  0.56  0.54  0.67 | .19  .07  -.01  .14  -.04  -.04  -.17 | 3.02  -0.10  0.88  2.09  -0.55  -0.56  -2.05 | **.01**  .47  .49  **.04**  .89  .99  **.05** |
| DV: ERN  Age  OCI-R  BDI-II  Risk for SUD  Risk for Anxiety  Risk for Depression  Risk for OCD  OCD x Depression  OCD x SUD  OCD x Anxiety  SUD x Anxiety  SUD x Depression  Anxiety x Depression | 0.10 | 2.51 | 13, 283 | <.01 | 0.06  0.01  0.03  1.18  -2.94  -0.41  -1.55  -0.80  -1.67  2.551.28  0.84  1.47 | 0.02  0.03  0.04  1.04  1.25  0.82  0.94  1.10  1.69  1.23  1.65  1.52  1.19 | .19  .01  .08  .12  -.34  -.05  -.19  -.08  -.13  .27  .09  .06  .14 | 2.61  0.89  0.60  1.45  -2.71  -0.08  -2.64  -1.01  -1.18  2.65  0.7  0.56  1.59 | **.01**  .96  .39  .27  **.008**  .60  **.04**  **.**43  .25  **.03**  .40  .54  .18 |

Note: DV, dependent variable; ERN, error-related negativity; OCI-R, Obsessive-Compulsive Inventory-Revised; BDI-II, Beck Depression Inventory-II; SUD, Substance Use Disorder; OCD, obsessive–compulsive disorder;

***SI 4: Associations between ERN and symptom measures as well as obsessive-compulsive symptom severity and symptom dimensions***

We have performed regression and correlational analyses to determine the relation between ERN and symptom measures in the whole sample as well as OCD symptom severity, symptom dimensions and clinical features in patients only. Table S5 presents correlations between ERN amplitudes and symptom measures in the whole sample. Table S6 shows associations between ERN and clinical measures in OCD patients. After correction for multiple comparisons only harm avoidance showed a significant association to error-related brain activity. Higher harm avoidant motivation was associated with more negative, i.e., larger amplitudes of the ERN (r=-.19, p<.001, see supplementary Figure 3). No other association between error-related brain activity and symptom measures in the whole sample were observed. Further, multiple regression analyses were performed for the OCD patient group analyzing the prediction of error-related brain activity by OCD symptom dimensions derived from the Y-BOCS Checklist (Katerberg *et al.*, 2010, see Table S7 and S8). Dimension scores of OCD symptoms were determined for each patient using the Y-BOCS checklist (Goodman *et al.*, 1989) according to a method described by Katerberg et al. (Katerberg *et al.*, 2010). The five dimensions were: taboo, contamination/cleaning, doubt, rituals/superstitious and hoarding/symmetry. None of the symptom dimensions neither lifetime nor current symptom expression was associated with error-related brain activity.

**Supplementary Table 5:** Correlations between error-related negativity and symptom measures in the whole sample.

|  | ERN  peak-to-peak | | ERN  area around peak | | Δ ERN  peak-to-peak | |
| --- | --- | --- | --- | --- | --- | --- |
| Symptom measures | r | p | r | p | r | p |
| Beck-Depression-Inventory II  Obsessive-Compulsive Inventory-Revised (OCI-R)  OCI-R washing  OCI-R checking  OCI-R ordering  OCI-R obsessing  OCI-R hoarding  OCI-R neutralizing  NEO-Five-Factor Inventory  Neuroticism  Extraversion  Obsessive-Compulsive Trait Core Dimensions Questionnaire  OC-TCDQ harm avoidant motivation  OC-TDCQ incompleteness, just right motivation  State-Trait Anxiety Inventory  State-Version  Trait-Version  Verbal IQ | -.05  -.05  -.06  -.08  -.01  -.10  -.04  .03  -.11  .09  -.13°  -.10  .00  -.06  -.04 | .41  .38  .31  .18  .83  .08  .55  .58  .06  .14  .03  .08  .99  .30  .49 | -.05  -.08  -.09  -.11  .007  -.12°  -.07  -.005  -.14°  .07  **-.19***  -.13°  -.05  -.09  -.05 | .39  .19  .12  .07  .91  .04  .23  .93  .02  .26  **.001**  .03  .41  .13  .44 | -.03  -.07  -.09  -.10  -.02  -.11  -.03  .005  -.08  .06  -.12°  -.10  -.01  -.04  -.01 | .61  .25  .12  .09  .76  .06  .62  .93  .15  .27  .04  .07  .86  .47  .82 |

Note: ° Significant correlation without correction for multiple comparisons. * Significant after correction for multiple comparisons. Significance level was adjusted to α = .003 to correct for multiple comparisons by Bonferroni’s correction*.*


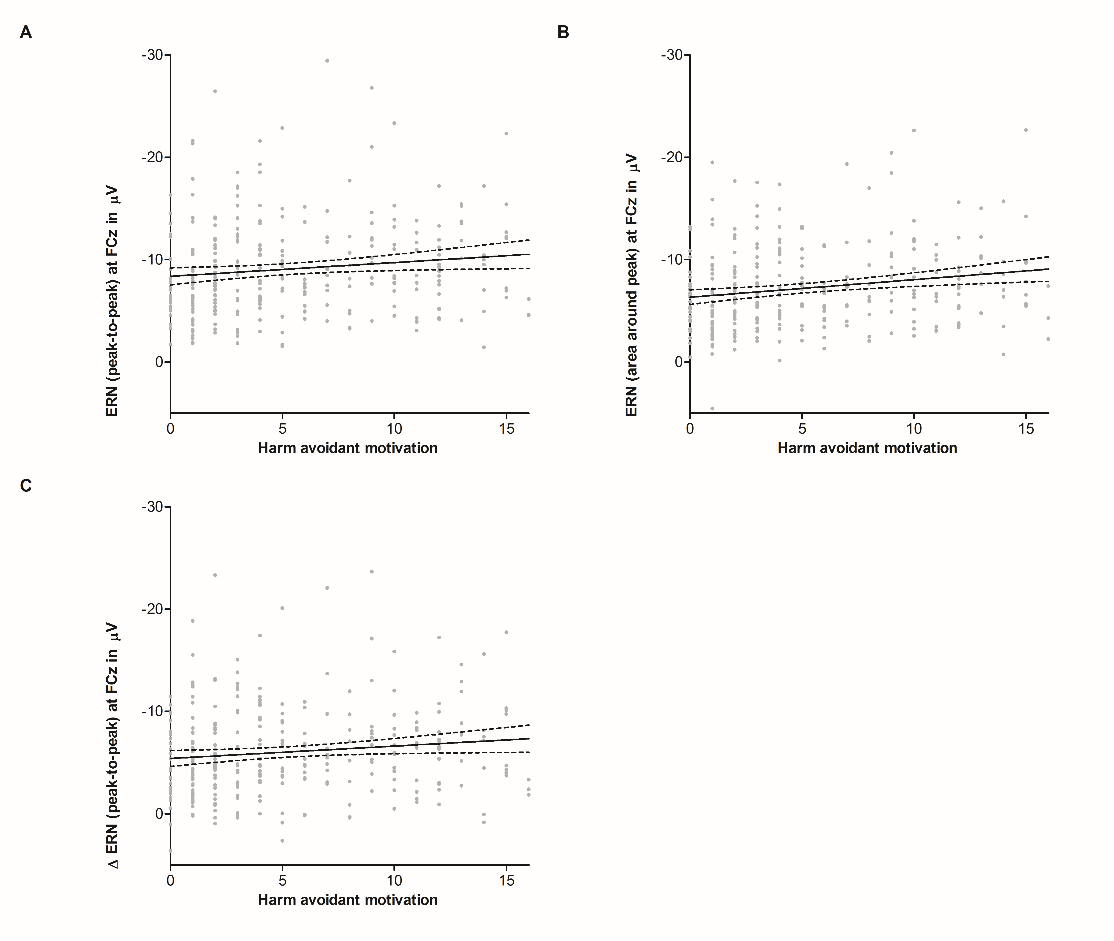


**Supplementary Figure 3**: Scatterplot depicting the correlations between ERN measured as peak-to-peak (A), as area around the peak (B), and ΔERN (C), and harm avoidant motivation as measured with OC-TCDQ.

**Supplementary Table 6:** Correlations between error-related negativity and clinical measures in OCD patients.

|  | ERN  peak-to-peak | | ERN  area around peak | | Δ ERN  peak-to-peak | |
| --- | --- | --- | --- | --- | --- | --- |
| Clinical measures | r | p | r | p | r | p |
| Yale-Brown Obsessive-Compulsive Scale (total score)  Yale-Brown Obsessive-Compulsive Scale (obsessions score)  Yale-Brown Obsessive-Compulsive Scale (compulsions score)  Age of onset OCD  Clinical Global Impression  Global Assessment of Functioning  Montgomery-Asberg Depression Rating Scale | -.11  -.13  -.05  .04  .07  -.05  -.02 | .24  . 18  . 59  .70  .47  .58  .80 | -.06  -.08  -.007  .05  .07  -.09  -.03 | .53  .36  .94  .58  .49  .37  .74 | -.07  -.10  -.003  .12  .08  -.12  -.02 | .48  . 30  . 97  .20  .38  .21  .84 |

**Supplementary Table 7:** Multivariate regression model for the error-related negativity (peak-to-peak) as dependent variable and symptom dimension scores derived from the Yale-Brown Obsessive-Compulsive Scale Checkliste as predictors.

|  | Regression | | | |  | | | |  |
| --- | --- | --- | --- | --- | --- | --- | --- | --- | --- |
|  | *R²* | B | B (SE) | β | | *t* | *p* | *F* |  |
| **CURRENT**  **ERN (peak-to-peak)**  Taboo  Contamination/cleaning  Doubts  Superstitions/rituals  Symmetry/hoarding | .03 | 2.09  -0.24  0.78  0.84  0.47 | 2.66  1.71  1.95  2.33  2.24 | .09  -.02  .05  .04  .03 | | 0.79  -0.14  0.40  0.36  0.21 | 0.43  0.89  0.69  .72  .83 | 0.55 |  |
| **LIFETIME**  **ERN (peak-to-peak)**  Taboo  Contamination/cleaning  Doubts  Superstitions/rituals  Symmetry/hoarding | .007 | 0.83  -0.34  0.80  0.25  -0.18 | 2.20  1.59  1.85  2.13  2.29 | .05  -.02  .05  .01  -.01 | | 0.38  -0.21  0.44  0.12  -0.08 | .71  .83  .67  .91  .94 | 0.16 |  |

Note: B (unstandardized coefficient), SE (standard error), β (standardized coefficient)

**Supplementary Table 8:** Multivariate regression model for the error-related negativity (area around peak) as dependent variable and symptom dimension scores derived from the Yale-Brown Obsessive-Compulsive Scale Checkliste as predictors.

|  | Regression | | | |  | | | |  |
| --- | --- | --- | --- | --- | --- | --- | --- | --- | --- |
|  | *R²* | B | B (SE) | β | | *t* | *p* | *F* |  |
| **CURRENT**  **ERN (area around peak)**  Taboo  Contamination/cleaning  Doubts  Superstitions/rituals  Symmetry/hoarding | .009 | 1.99  -0.19  -0.60  -.60  1.06 | 2.37  1.53  1.74  2.08  2.0 | .10  -.01  -.05  -.03  .06 | | 0.84  -0.12  -0.35  -.29  .53 | .40  .90  .73  .77  .60 | 0.20 |  |
| **LIFETIME**  **ERN (area around peak)**  Taboo  Contamination/cleaning  Doubts  Superstitions/rituals  Symmetry/hoarding | .014 | 1.65  -0.40  -1.39  -0.25  -0.43 | 1.95  1.4  1.63  1.88  2.02 | .10  -.03  -.11  -.02  -.03 | | 0.85  -0.28  -0.85  -0.13  -0.21 | .40  .78  .39  .89  .83 | 0.31 |  |

Note: B (unstandardized coefficient), SE (standard error), β (standardized coefficient)

***SI 5: Heritability Analyses***

Heritability estimates (h²_r_) were obtained using the Sequential Oligogenic Linkage Analysis Routines (SOLAR) Version 8.3.1 (<https://www.nitrc.org/projects/se_linux>). The SOLAR software integrates a pedigree-based variance-component and multipoint identity-by-descent probability calculations to compare the null hypothesis of no heritability to a model which estimates the phenotypic variance that is explained by additive genetic factors (Almasy and Blangero, 1998). In order to increase the power of the heritability analyses we added patients with OCD and unaffected first-degree relatives from a previous study that used the same methods (Riesel *et al.*, 2011). The presented analyses is based on 325 participants including 125 patients, 70 relatives, and 130 comparison participants. Age, gender and the interaction between age and gender were entered as covariates to the model and those showing a significant association with ERN were retained in the heritability analysis. The heritability estimates were presented in Table S9. The highest heritability estimate has been observed for the differences between ERN and CRN. Approximately half of the variance of this measure of error-related brain activity can be attributed to additive genetic factors. This is in line to heritability estimates derived from a previous twin study (Anokhin *et al.*, 2008). ERN amplitude reached a heritability of. 32 and does not reach significance which is related to the limited power in our sample, that has a fairly small sample size for heritability analyses. This is below the expected 50% (Anokhin *et al.*, 2008). However, previous studies repeatedly reported lower heritability estimates derived from family studies (Greenwood *et al.*, 2016) and heritability estimates vary substantially depending whether the source of calculations are twin, family or population based studies (Light *et al.*, 2014). Finally, unaffected family members not only have an increased risk for OCD but also for other associated disorders (Bienvenu *et al.*, 2011). The exclusion of first-degree relatives with mental disorders may have resulted in a biased “healthier” sample of less genetically vulnerable individuals and may have lowered the observed heritability estimates.

**Supplementary Table 9:** Heritability estimates.

|  |  |  |  | Covariate *p-*value | | |
| --- | --- | --- | --- | --- | --- | --- |
| Measure | h²_r_ | *SE* | p-value | age | Sex | Age x sex |
| Error-related negativity (peak-to-peak) | 0.22 | 0.27 | 0.21 | **<.0001** | .22 | **.03** |
| Error-related negativity (area around peak) | 0.32 | 0.29 | 0.14 | **<.0001** | .74 | **.01** |
| Δ Error-related negativity (area around peak) | **0.48** | 0.24 | **.03** | **<.0001** | **.05** | **.05** |

Significant heritability estimates and covariates are marked in bold. h²_r_ residual heritability after adjustment for significant covariates, SE = standard error

***Supplementary References***

**Almasy, L. & Blangero, J.** (1998). Multipoint quantitative-trait linkage analysis in general pedigrees. *American Journal of Human Genetics* **62**, 1198-211.

**Anokhin, A., Golosheykin, S. & Heath, A.** (2008). Heritability of frontal brain function related to action monitoring. *Psychophysiology* **45**, 524-34.

**Bienvenu, O. J., Samuels, J. F., Wuyek, L. A., Liang, K. Y., Wang, Y., Grados, M. A., Cullen, B. A., Riddle, M. A., Greenberg, B. D., Rasmussen, S. A., Fyer, A. J., Pinto, A., Rauch, S. L., Pauls, D. L., McCracken, J. T., Piacentini, J., Murphy, D. L., Knowles, J. A. & Nestadt, G.** (2011). Is obsessive-compulsive disorder an anxiety disorder, and what, if any, are spectrum conditions? A family study perspective. *Psychological Medicine*, 1-13.

**Goodman, W. K., Price, L. H., Rasmussen, S. A., Mazure, C., Fleischmann, R. L., Hill, C. L., Heninger, G. R. & Charney, D. S.** (1989). The Yale-Brown Obsessive Compulsive Scale. I. Development, use, and reliability. *Archives of General Psychiatry* **46**, 1006-11.

**Greenwood, T. A., Light, G. A., Swerdlow, N. R., Calkins, M. E., Green, M. F., Gur, R. E., Gur, R. C., Lazzeroni, L. C., Nuechterlein, K. H., Olincy, A., Radant, A. D., Seidman, L. J., Siever, L. J., Silverman, J. M., Stone, W. S., Sugar, C. A., Tsuang, D. W., Tsuang, M. T., Turetsky, B. I., Freedman, R. & Braff, D. L.** (2016). Gating Deficit Heritability and Correlation With Increased Clinical Severity in Schizophrenia Patients With Positive Family History. *American Journal of Psychiatry* **173**, 385-91.

**Katerberg, H., Delucchi, K. L., Stewart, S. E., Lochner, C., Denys, D. A., Stack, D. E., Andresen, J. M., Grant, J. E., Kim, S. W., Williams, K. A., den Boer, J. A., van Balkom, A. J., Smit, J. H., van Oppen, P., Polman, A., Jenike, M. A., Stein, D. J., Mathews, C. A. & Cath, D. C.** (2010b). Symptom dimensions in OCD: item-level factor analysis and heritability estimates. *Behavior Genetics* **40**, 505-517.

**Lee, M., Rebora, P., Valsecchi, M. G., Czene, K. & Reilly, M.** (2013). A unified model for estimating and testing familial aggregation. *Statistics in Medicine* **32**, 5353-5365.

**Light, G., Greenwood, T. A., Swerdlow, N. R., Calkins, M. E., Freedman, R., Green, M. F., Gur, R. E., Gur, R. C., Lazzeroni, L. C., Nuechterlein, K. H., Olincy, A., Radant, A. D., Seidman, L. J., Siever, L. J., Silverman, J. M., Sprock, J., Stone, W. S., Sugar, C. A., Tsuang, D. W., Tsuang, M. T., Turetsky, B. I. & Braff, D. L.** (2014). Comparison of the heritability of schizophrenia and endophenotypes in the COGS-1 family study. *Schizophrenia Bulletin* **40**, 1404-11.

**Riesel, A., Endrass, T., Kaufmann, C. & Kathmann, N.** (2011). Overactive Error-Related Brain Activity as a Candidate Endophenotype for Obsessive-Compulsive Disorder: Evidence From Unaffected First-Degree Relatives. *American Journal of Psychiatry* **168**, 317-324.
